# Old age liaison psychiatry: audit assessing adherence to referral pathway and referral characteristics including indications, interventions and outcomes

**DOI:** 10.1192/bjo.2021.330

**Published:** 2021-06-18

**Authors:** Alfred Wong, Kimberley Boyle

**Affiliations:** 1 Dumbarton Joint Hospital; 2 Glasgow Royal Infirmary

## Abstract

**Aims:**

This audit covered 3 hospitals in Glasgow City which has 1221 beds providing inpatient healthcare for the north east region of the city. To improve the referral process,we aimed to verify adherence to existing referral pathway and adequacy of information provided by referrals. Referral characteristics including referral indication, intervention and outcomes were accounted for to identify area interest that may help improve the referral process.

**Method:**

Our referral pathway involves completion of a Microsoft Word referral template subsequently sent electronically to an internal electronic mail.

Referrals in a 2 month period were included in the audit. Each referral was reviewed for adherence to the referral template, adequacy of provided information and referral indications. Intervention in the form of staff input, Mental Health Act status, psychotropic medication prescribed and given diagnosis was ascertained via staff electronic entry records.

**Result:**

139 referrals were included. 114 referrals (82%) adhered to the referral template. 72 referrals (52%) contained adequate information. Common referral indications were delirium (23%), agitation (20%), low mood (18%) and cognitive decline queries (18%). Staff input ranged from psychiatrist input (46%), liaison nurses (40%), clinical psychology (1%) and shared input (13%). 16 referrals (12%) resulted in subsequent detention under the Mental Health Act. Psychotropic medications prior to liaison assessment included antidepressants (49%), antipsychotics (29%) and benzodiazepines (16%). Liaison assessment resulted in increase use of antipsychotic (55%) and reduction of antidepressants (29%) and benzodiazepines (10%), Delirium (34%), dementia (21%), Mood & Anxiety related disorders (18%) and Query of Cognitive Impairment (14%) were recorded as the most discussed diagnosis.

**Conclusion:**

Referrals with inadequate details affect the service's ability to efficiently assess for clinical urgency and matching of appropriate interventions to suit clinical needs. The percentage difference in delirium between referral indication and diagnosis highlights that delirium can be under-recognised, resulting in potentially delayed treatment. Identifying common given diagnosis and differences in psychotropic medication prescribing pattern points to the need for training and support of acute medical ward staff in utilising therapeutics for management of acute mental health disorder.

A pending electronic referral pathway with mandatory entries and linked relevant online resources can encourage early recognition of acute mental health disorder and prompt early management including the use of appropriate therapeutics. An additional feature allowing direct referrals by acute ward staff to community mental health team would support continuity of care for discharged patients needing ongoing mental health assessment.

